# Experiences of stigma and discrimination faced by family caregivers of people with schizophrenia in India

**DOI:** 10.1016/j.socscimed.2017.01.061

**Published:** 2017-04

**Authors:** Mirja Koschorke, R. Padmavati, Shuba Kumar, Alex Cohen, Helen A. Weiss, Sudipto Chatterjee, Jesina Pereira, Smita Naik, Sujit John, Hamid Dabholkar, Madhumitha Balaji, Animish Chavan, Mathew Varghese, R. Thara, Vikram Patel, Graham Thornicroft

**Affiliations:** a Centre for Global Mental Health, Institute of Psychiatry, Psychology and Neuroscience, King's College, London, UK; b Schizophrenia Research Foundation (SCARF), Chennai, India; c Samarth, Chennai, India; d Centre for Global Mental Health, London School of Hygiene and Tropical Medicine, London, UK; e MRC Tropical Epidemiology Group, London School of Hygiene and Tropical Medicine, London, UK; f Sangath, Goa, India; g Parivartan, Satara, India; h Nirmittee, Satara, India; i NIMHANS, Bengaluru, India

**Keywords:** Stigma, Discrimination, Knowledge, Schizophrenia, Mental illness, Caregiver, Family, India

## Abstract

Stigma associated with schizophrenia significantly affects family caregivers, yet few studies have examined the nature and determinants of family stigma and its relationship to their knowledge about the condition. This paper describes the experiences and determinants of stigma reported by the primary caregivers of people living with schizophrenia (PLS) in India. The study used mixed methods and was nested in a randomised controlled trial of community care for people with schizophrenia. Between November 2009 and October 2010, data on caregiver stigma and functional outcomes were collected from a sample of 282 PLS–caregiver dyads. In addition, 36 in-depth-interviews were conducted with caregivers. Quantitative findings indicate that ‘high caregiver stigma’ was reported by a significant minority of caregivers (21%) and that many felt uncomfortable to disclose their family member's condition (45%). Caregiver stigma was independently associated with higher levels of positive symptoms of schizophrenia, higher levels of disability, younger PLS age, household education at secondary school level and research site. Knowledge about schizophrenia was not associated with caregiver stigma. Qualitative data illustrate the various ways in which stigma affected the lives of family caregivers and reveal relevant links between caregiver-stigma related themes (‘others finding out’, ‘negative reactions’ and ‘negative feelings and views about the self’) and other themes in the data.

Findings highlight the need for interventions that address both the needs of PLS and their family caregivers. Qualitative data also illustrate the complexities surrounding the relationship between knowledge and stigma and suggest that providing ‘knowledge about schizophrenia’ may influence the process of stigmatisation in both positive and negative ways. We posit that educational interventions need to consider context-specific factors when choosing anti-stigma-messages to be conveyed. Our findings suggest that messages such as ‘recovery is possible’ and ‘no-one is to blame’ may be more helpful than focusing on bio-medical knowledge alone.

## Highlights

• Experiences of being blamed and worries about marital prospects were salient.
• Caregivers' and PLS' experiences of stigma were determined by similar factors.
• Knowledge was linked to stigma in qualitative but not in quantitative analyses.
• Context-specific messages rather than biomedical knowledge may help reduce stigma.
• Family caregivers need access to emotional and social support in their own right.

## 1 Introduction

Problems related to stigma do not only affect persons suffering from mental illness but also families (Corrigan et al., 2006, Phelan et al., 1998). In his seminal work on stigma in the 1960s, Goffman already reflected upon the stigma that spills over to families, coining the term ‘courtesy stigma’ (Goffmann, 1963). The negative impact of this form of stigma (which we will refer to as ‘family stigma’) may be particularly marked in settings where family cohesion is high. In India, as in many low and middle-income countries (LAMIC), most people living with schizophrenia (PLS) live with their families and rely on them for both economic support and everyday care (Thara, 1993). Thus, family members are closely involved in most aspects of PLS’ care and often maintain control of help-seeking and treatment decisions, assuming many of the roles filled by health or social care staff in high-income country (HIC) settings (Nunley, 1998). The high quality of family support provided to many PLS in India is likely to reflect a widely held social norm that no one should have to live alone because of their illness (Thara, 1993).

At the same time, studies in India also document how, in the absence of adequate health and social care, particularly in life domains such as finances, family relationships, well-being and health, family members of PLS cope with enormous caregiver burden (Kumar et al., 2015). Stigma adds to the burden of caregiving and affects the lives of family members of PLS in multiple ways. For example, research from South India has found that family caregivers of PLS were often concerned that other family members would not be able to marry or that friends, relatives or neighbours might avoid or treat them differently (Raguram et al., 2004, Thara et al., 2003, Thara and Srinivasan, 2000). Similar findings have been reported for other LAMIC (Chien et al., 2014, Phillips et al., 2002, Shibre et al., 2001) and several HIC (Larson and Corrigan, 2008).

Lack of knowledge about mental illness has been described as one of the components of the stigma construct itself, for example in Thornicroft's conceptualisation of stigma as an overarching construct consisting of problems of knowledge (ignorance), attitudes (prejudice) and behaviour (discrimination) (Thornicroft, 2006). Many anti-stigma interventions aim to improve knowledge about mental illness (Mehta et al., 2015) and health care interventions for family members of PLS often focus on ‘knowledge about schizophrenia’ (Sin and Norman, 2013). Poorer knowledge about mental illness has been linked to stigmatising attitudes in several studies (Jorm et al., 2006, Thornicroft, 2006), but little is known about the links between knowledge about mental illness and subjective stigma experience, particularly among family members. A better understanding of this relationship may inform efforts to reduce the impact of stigma, for example by suggesting messages to be conveyed in educational interventions (Clement et al., 2010).

Findings on the experiences of stigma of PLS taking part in this study have been reported previously in this journal (Koschorke et al., 2014). The aim of the present paper is to describe caregivers’ own experiences of stigma, and the factors influencing these experiences in India. We also examine the hypothesis that caregivers with lower levels of knowledge about schizophrenia experience higher levels of stigma.

## 2 Methods

### 2.1 Setting

The study was nested in a randomised controlled trial of collaborative community care for PLS in India (COPSI trial) which was implemented in three diverse settings: in rural Tamil Nadu by the Schizophrenia Research Foundation (SCARF), and in two mixed urban and rural sites, Goa and Satara (Maharashtra), by the NGOs ‘Sangath’, ‘Parivarthan’ and ‘Nirmittee’ in collaboration with private psychiatrists. Methods and findings of the COPSI trial have been described elsewhere (Balaji et al., 2012a, Balaji et al., 2012b, Chatterjee et al., 2015, Chatterjee et al., 2011, Chatterjee et al., 2014). The nested study on stigma used cross-sectional data collected at the point of entry into the trial and employed a mixed-methods approach, combining quantitative data from all PLS and caregivers in the trial and qualitative data from a purposively selected subsample of PLS and caregivers. The methods used have been described in detail in our publication on PLS’ experiences of stigma (Koschorke et al., 2014), and will therefore only be summarised briefly here.

### 2.2 Recruitment and sampling

The quantitative sample for the study comprised all PLS and caregivers recruited for the COPSI trial (n = 282 PLS-caregiver dyads). Eligibility criteria for PLS were: i) age 16–60 years; ii) a primary diagnosis of schizophrenia by ICD-10 DCR criteria (WHO, 1992); iii) illness duration of at least 12 months and an overall moderate severity of the illness based on the Clinical Global Impression-Schizophrenia(CGI-SCH) scale rating (Haro et al., 2003); and iv) residing within the study catchment area for the duration of the study. One primary caregiver (usually the family member most closely involved with the PLS in everyday life) was recruited for each PLS.

For the qualitative study component, a purposive sampling technique was utilised in an effort to ensure adequate sample variability for PLS gender, severity of illness according to the PANSS (Kay et al., 1987), highest education level in the household and research site. In order to facilitate the in-depth study of experiences of stigma and discrimination, there was oversampling of dyads in which PLS reported higher levels of negative discrimination according to the DISC negative discrimination scale (Thornicroft et al., 2009) Overall, 36 PLS-caregiver dyads were recruited to allow for adequate numbers in each sampling category.

### 2.3 Data collection

Quantitative data on caregiver stigma were collected using an adapted version of the stigma section of the Family Interview Schedule, which had been developed for the International Study of Schizophrenia (Sartorius and Janca, 1996) and previously used in a similar population in India (Thara and Srinivasan, 2000). It comprised of 10 items on stigma experience (e.g. *‘you worried that your neighbours would treat you differently’*) that were scored from ‘not at all’ to ‘a lot’ (scores 0–3). In addition, caregivers rated their willingness to disclose their relative's illness on a single item scored on a Likert scale ranging from 1-5 (*‘In general, how comfortable would you feel talking to a friend or family member about your ill family member's mental health, for example telling them he/she has a mental health diagnosis and how it affects him/her and the family?’*), adapted from a similar item for people with mental illness (Koschorke et al., 2014, TNS UK Care Services Improvement Partnership, 2009). Caregivers knowledge about schizophrenia was measured using the Knowledge About Schizophrenia Interview (KASI) (Barrowclough et al., 1987), which assesses six domains of understanding: Knowledge about diagnosis, symptomatology, aetiology, medication, course and prognosis and management.

A standardised process of translation and validation of tools was followed, as has been described previously (Chatterjee et al., 2011, Chatterjee et al., 2014). Measures on stigma underwent an additional process of validation through focus group discussions involving PLS, caregiver and mental health staff representatives. Three items of the Family Interview Schedule (two on coping strategies and one on general illness impact) were removed to ensure all items used related directly to experiences of stigma.

Data collection took place between November 2009 and October 2010, prior to trial procedures starting. Quantitative data was collected using programmed palmtop computers and pen and paper methods. In addition, semi-structured in-depth-interviews were carried out with a subsample of the PLS and caregivers recruited for the trial.

The guide for qualitative interviews was developed in a collaborative, step-wise process involving research teams and local clinicians in all study sites and was tested and adapted in a series of 27 formative and 24 pilot interviews (Balaji et al., 2012a, Balaji et al., 2012b). Ongoing data analysis served to continually refine the phrasing of interview questions and probes.

Interviews were held in the local languages of the sites (Konkani, Marathi or Tamil) or in English, as preferred by the participants. Care was taken to carry out interviews in private, either in the family's home or at another location nearby. Interviews were audio-recorded using digital voice recorders, after receiving permission from participants.

Ethics approval was obtained from the Institutional Review Boards at SCARF and Sangath in India and the Ethics Committees at the London School of Hygiene and Tropical Medicine (approval number 5579) and King's College, London (PNM/08/09–121) in the UK.

### 2.4 Analysis

Statistical analyses were conducted using Stata 11 (Stata Corp, 2009). To test the hypothesis that higher caregiver experience of stigma was associated with higher knowledge about schizophrenia, linear regression was used to assess the association of the Caregiver Stigma Mean Score (CSMS) expressed as a continuous outcome with the Knowledge about Schizophrenia Interview (KASI) total score and each of its sub-scores, expressed as categorical variables. Given the lack of any crude correlations, no further analytic steps were carried out, and findings were interpreted with regard to the hypothesis.

Next, we aimed to identify factors independently associated with caregiver stigma. First, the CSMS was examined in relationship to socio-demographic and clinical variables in univariate analyses using linear regression. Potential predictors were then identified using an analytic diagram. Factors associated with a significant (p < 0.1) outcome were included in the multivariable linear regression model following a hierarchical approach (Victora et al., 1997). After adjusting for the other factors in the model, only those factors which remained significant (p < 0.1) were retained in the final model. The analysis of qualitative data used techniques of thematic analysis (Braun and Clarke, 2006) and was carried out during the process of data collection. Qualitative analysis software (NVivo 8 (QSR International Pty Ltd., 2008)) was used for coding, as well as for higher levels of analysis.

The analytic process was collaborative and involved the authors and interviewers in all study sites. The method employed a combination of deductive and inductive principles. The list of topics covered by the interview guide was derived from a deductive framework based on a literature review, which was set aside once data collection had commenced to allow the process to be as inductive as possible. Thus, analysis did not follow a specific stigma framework but rather aimed to explore the meaning of ‘stigma’ from the perspective of interviewees. First, a set of transcripts were coded independently by researchers using ‘open coding’(Green and Thorogood, 2004). The group discussed the codes and tentative categories were derived. A further six interviews were coded independently by RP and MK using the revised scheme. Coding was compared, significant differences resolved and definitions clarified.

MK then coded a representative subset of transcripts (n = 24 interview pairs) while 12 interview pairs were coded by RP and SK. The scheme was continually developed as analysis progressed to incorporate new codes. A trail of coding steps was maintained.

The process of identification of themes and links in the data started during coding and involved a collaborative review of the material as well as clustering of sub-codes and codes to form categories. Relationships between subcategories and categories were examined in order to decide which categories informed the same overarching concepts. Preliminary themes were established and examined for internal homogeneity and external heterogeneity (Braun and Clarke, 2006). PLS and caregiver narratives were first analysed in parallel, in order to triangulate the study findings on PLS’ experiences of stigma from both sources (Koschorke et al., 2014), for example, the study would look at negative societal reactions faced by PLS, both from the perspective of their own reports and from the perspective of primary caregivers. The second phase of analysis focused on caregivers' own experiences of stigma. Preliminary categories and themes were reviewed and validated and additional themes relevant to caregiver's experiences identified.

The development of the thematic network was inductive and drew upon tentative links among categories and themes which were captured while coding using a level of inductive codes that captured statements in which these links were apparent. It was further informed by a log of analytic observations noted during coding, case summaries and analytic collaborator's meetings. A preliminary thematic network was drawn up based on an interim analysis of 24 caregiver interviews. It was then cross-checked and validated based on the full data set available (n = 36). This additional step of validation did not yield any significantly different findings, but added further detail and depth to the analysis. The final thematic network presented in Fig. 2 illustrates the results of this substantially data-driven process.

## 3 Results

### 3.1 Sample description

Table 1 describes characteristics of caregivers and key characteristics of PLS. Of the 282 PLS-caregiver dyads, caregivers were predominantly female (67%), with a mean age of 49 years (range 17–85 years) (Table 1). They generally related to the PLS as a parent (51%) or spouse (25%). 45% of caregivers had completed 9 or more years of schooling, and 50% were pursuing an income-generating occupation. Full sample characteristics can be accessed in Online File A, Table 1.

The 36 PLS-caregiver dyads who took part in the qualitative component comprised 18 male and 18 female PLS with 12 male and 24 female caregivers. Dyads were distributed proportionally to the quantitative sample across the three sites, with 14 dyads from Tamil Nadu, 10 from Satara and 8 from Goa. The qualitative sub-sample was similar to the quantitative sample with regards to key socio-demographic characteristics, symptom severity and disability levels. Characteristics of the qualitative sample can be accessed in Online File A, Table 2.

### 3.2 Caregivers’ experiences of stigma

This section presents the quantitative findings on caregiver stigma, followed by the results of qualitative analyses. In the discussion section, the findings of both methodologies are integrated and discussed, whereby qualitative findings are used to contextualise and triangulate the quantitative findings obtained.

#### 3.2.1 Quantitative findings

**Fig. 1:**
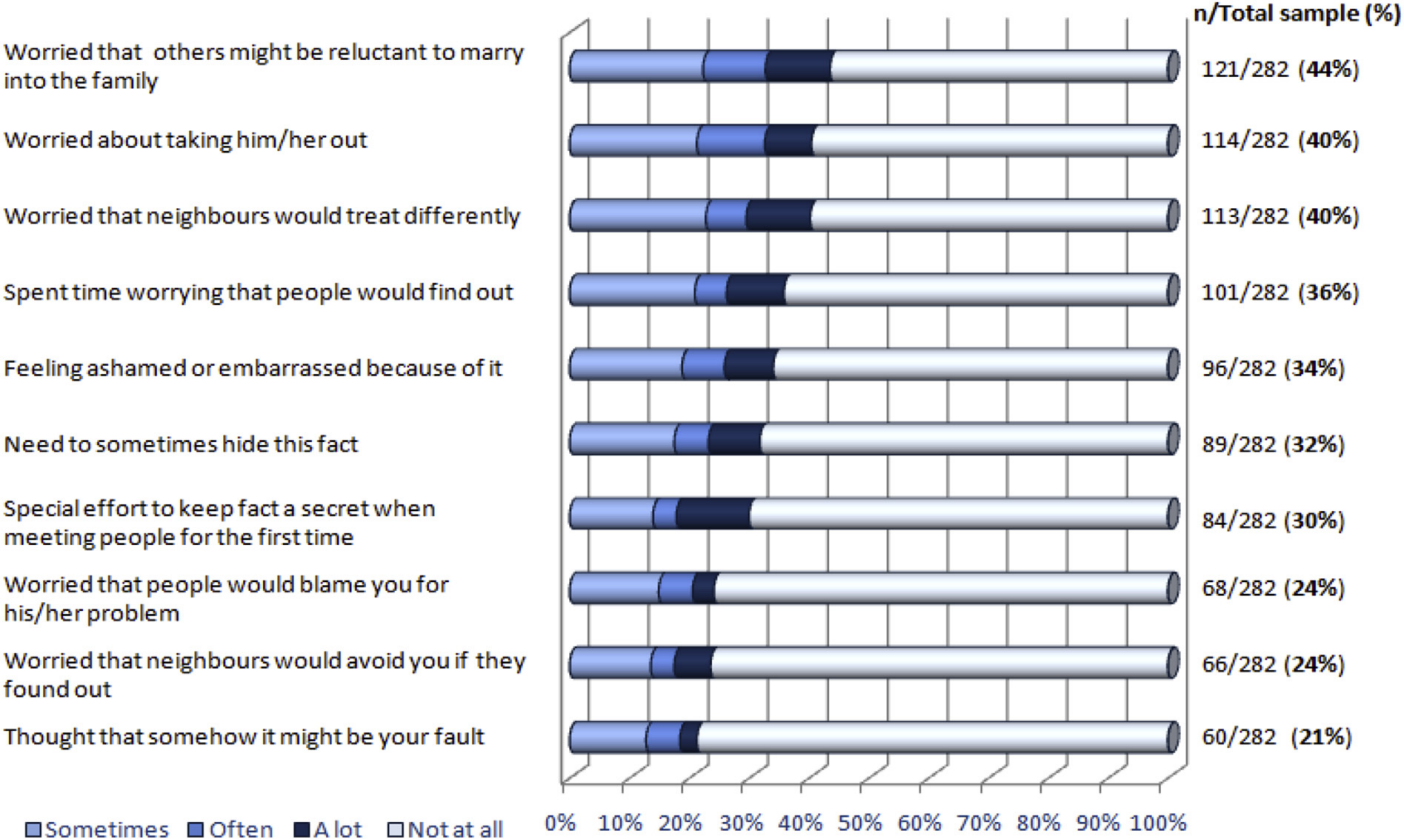
Caregiver stigma item percentages.

The median Caregiver Stigma Mean Score (CSMS) was 0.4 (range 0–3) with an inter-quartile range of 0.1–0.9. About 1 in 5 caregivers (n = 59; 21%) had experienced ‘high caregiver stigma’ (each item score ≥1) in the last 12 months. The proportion reporting stigma on at least one item was 83% (n = 233), which also means that 17% (n = 79) did not report any stigma in the last year. Fig. 1 indicates the proportion of caregivers who endorsed each item of the caregiver stigma scale.

On the item on willingness to disclose the illness, 45% of caregivers indicated that they felt ‘uncomfortable’ or ‘very uncomfortable’ to tell others about their family member's mental health problem. 48% said they were ‘comfortable’ or ‘very comfortable’.

Caregivers' knowledge about schizophrenia was assessed using the Knowledge about Schizophrenia Interview (KASI) (Barrowclough et al., 1987), which has been used in LAMIC previously (Li and Arthur, 2005). The mean KASI total score in this study was 13.4. This means that participants on average scored 2.2 points on each item of the 4 point Likert scale, wherein a score of 2 indicates ‘little or no understanding to a level that is not necessarily detrimental’ and 3 indicates ‘good understanding’. The ranking on the item sub-scores (possible range 1–4; 4 indicating higher knowledge) was as follows: Knowledge about management (mean item score: 2.8); Knowledge about symptomatology (2.3); Knowledge about medication (2.3); Knowledge about diagnosis (2.1) and Knowledge about course and prognosis of schizophrenia (1.9) (see Table 1 Online File A).

In linear regression, no association was found between caregiver stigma experience (CSMS) and caregiver knowledge about schizophrenia (KASI Total Score) (p = 0.67) or between caregiver stigma experience and any of the KASI sub-scores (see Table 3 Online File C).

Multivariate models to determine the factors independently associated with caregiver stigma led to the following conclusions: Higher caregiver stigma experience was independently associated with higher PANSS positive symptom score (p = 0.003), higher levels of disability (p = 0.04), lower PLS age (p = 0.003), the highest education level in the household being secondary school level (9th to 12th Standard) (p = 0.03), and research site (Goa had the highest caregiver stigma rates, followed by Tamil Nadu and then Satara; p = 0.03). Crude and adjusted regression coefficients are given in Table 4 Online File D.

#### 3.2.2 Qualitative findings

Seven themes emerged from the qualitative analysis of caregiver interview data. Four of these mirror domains already identified for PLS’ experiences of stigma (Koschorke et al., 2014) but reflect the perspectives of caregivers (rather than those of PLS). Three themes relate specifically to caregivers' own experiences of stigma, the subject of this study, and will therefore be the focus of this report (‘key themes’).

• Key theme 1: *‘Others finding out – caregiver perspective’*
• Key theme 2: *‘Negative reactions towards the caregiver and changes in relationships’*
• Key theme 3: *‘Caregivers’ emotional reactions and feelings about the self’*

The findings on the other four themes identified are described to the extent required to illustrate how they were linked to each of the three key themes.

The themes were:

• *‘Behaviours and manifestations of the illness – caregiver perspective’*
• *‘PLS’ reduced ability to meet role expectations and personal aims – caregiver perspective’;*
• *‘Caregivers’ reduced ability to meet role expectations and personal aims’*
• *‘Negative reactions towards the PLS and changes in relationships – caregiver perspective’*

**Fig. 2:**
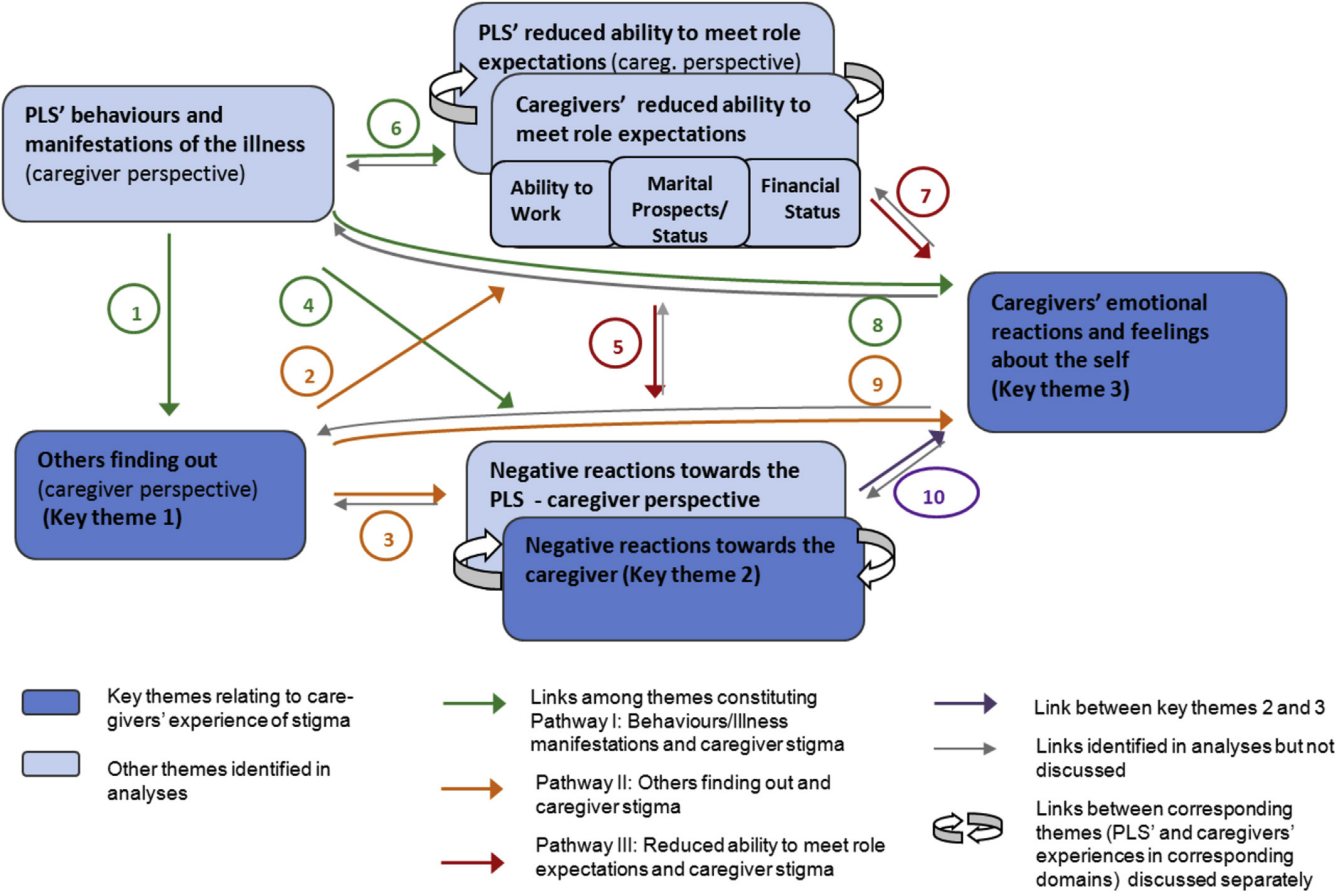
Thematic network caregivers' experiences of stigma.

All themes and links among them are summarised in a ‘thematic network’ (Attride-Stirling, 2001) in Fig. 2. We further describe findings on the contextual domain *‘caregivers’ understanding of the PLS’ illness’* to provide context to quantitative findings on ‘knowledge about schizophrenia’.

##### Key theme 1: ‘Others finding out’

Whether and how much other people knew about the PLS’ illness was often a matter of great concern to caregivers. Most felt that other people should not know as they feared negative consequences for the PLS, themselves and other family members. Caregivers often felt responsible for having to manage information about the PLS’ condition in the interest of the family, and were concerned that some of the PLS’ ‘behaviours and illness manifestations’, particularly poor self-care, inappropriate dress or side effects of medication might lead to ‘others finding out’ (see *Arrow 1* Fig. 2). Pervasive consequences to illness disclosure were anticipated, particularly in those families that had managed to conceal it. Caregivers' concerns for the PLS were similar to those that PLS had for themselves (Koschorke et al., 2014) e.g., worries that other people might look down upon the PLS, or treat them without respect. They further feared negative consequences for *themselves or other family members*, particularly *blaming* and *avoidance* (*Arrow 2*). Another common worry was that others might *gossip* and *‘spread the news’*.

Their greatest concern was, however, the impact that disclosure would have on the PLS’ ability to meet *role expectations in life areas of social salience, particularly marital prospects* (see also (Koschorke et al., 2014)). At the same time, caregivers saw their own and other family members' prospects for marriage and respect by their in-laws threatened by ‘others finding out’ *(Arrow 3)*.

*If they are getting me married and if the person who is marrying me feels that my mother [PLS] is like this, I would feel bad (…). They may ill-treat me and dominate me … I fear for that*.

Female caregiver, daughter of female PLS, Tamil Nadu

Some narratives suggest that *‘others finding out’* was also a concern because it was in itself a painful experience as caregivers felt forced to publicly accept a perceived failing and lowered social status. The ensuing feelings of devaluation, the stress of secrecy and constant worries about what *might* happen were important factors linked to *caregivers' negative feelings and views about themselves* (*Arrow 9*).

Accordingly, many caregivers made *efforts to avoid others finding out about the PLS’ problem.* Several said they did not tell others or gave only very general information. Some attempted to remove PLS from situations in which they would interact with others, e.g., not taking them to social functions or telling them not to speak to the neighbours. Many caregivers *avoided social interactions generally* for fear of *being asked uncomfortable questions, or* facing *negative reactions*. A further strategy was to try and *influence the PLS’ behaviour*:

*[I feel that others should not know] because they will start saying that if she has a mental illness then why did we bring her [into the family] as our daughter-in-law? (…) Relatives and neighbours should not know; she needs to behave properly. (…) [I] tell her how to behave, [but] she does not realise, she speaks loudly or if she goes to attend any function, she hurries, I tell her (…) not to behave like that. (…) I make her understand that she should do work which is told to her (…) [When guests come,] I tell her not to come to the front often at that time, [to] stay in the kitchen and ask others to serve tea or breakfast for the guests.*

Female caregiver, mother-in-law of female PLS, Satara

Contrary to the above, some caregivers said they did not attempt to hide the illness, either because everyone already knew or because they could not hide it even if they wanted to. Some thought it was acceptable for other people to know, implying that the community at large was supportive. A few even felt that it could be helpful to tell others to get their support or gather information about available treatments. Several positive responses were reported to active disclosure to trusted individuals.

##### Key theme 2: Negative reactions towards the caregiver

Caregivers' reported a complex interplay of both positive and negative reactions from others. The most commonly cited negative reaction was *‘blaming’*. Caregivers reported they were blamed and criticised for the PLS’ behaviour, but also for *‘delivering a mad person’*, causing the PLS’ problems, e.g. by *‘pampering’* him or *not taking care of him properly*, for *not marrying him/her off* or *making sure he goes for work*, or *for not noticing the problem before bringing the PLS into the family as a daughter-in-law*.

*They would say that I have made him like this (…). They would say that he has been roaming around as mad and I am not taking care of him. When they speak like this, I feel very bad. (…)*

*They criticise me. They criticise me even now for his behaviour.*

Female caregiver, wife of male PLS, Tamil Nadu

Some caregivers experienced great distress at being ‘*aware that others think or speak badly about the PLS’*. In one extreme case, a female caregiver had been *asked* by the other villagers *to kill her own daughter*, who had been aggressive to her in an episode of acute illness, and who was untreated at the time as her mother had run out of money to pay for her treatment.

Several caregivers also reported *‘being avoided or excluded from social interactions’*; people stopped visiting them, did not invite them to functions or had stopped talking to them altogether. Sometimes, this appeared to be because they wanted to avoid the PLS rather than the caregiver per se. A few caregivers felt that they were *‘treated differently’* or *‘not respected’* by friends, neighbours or work colleagues (see Additional Quotes Online File E).

Caregivers also experienced both positive and negative reactions from other members of the family. Several faced *critical comments* or *disagreements* about how to manage the PLS’ behaviour and treatment, or tried to defend the interest of the PLS against other family members who were affected by him/her not working or getting married. Several caregivers *felt alone* in their caregiving role.

Most negative reactions that caregivers faced were linked to the ‘*PLS's behaviour and certain forms of manifestations of his or her illness’ (Arrow 4;* Fig. 2), particularly odd, disruptive or aggressive behaviour and poor self-care (see Additional quotes Online File D).

Caregivers were also *blamed* when PLS did not meet role expectations in socially salient life areas, e.g., they were criticised for *‘not discharging their duty’* as parents to get their child married, or for *‘pampering’* their son and not trying hard enough to get him/her to work *(Arrow 5)* (see Additional Quotes Online File D). In addition, caregivers' social relationships were affected by their own reduced ability to work and lowered financial status. In fact, caregivers often attributed *avoidance, distancing, critical comments or ‘being looked down upon’* not to the label of the illness per se, but the reduced social status of the family as a consequence of the PLS’ condition *(Arrow 5).* Caregivers' ability to work was impaired by the need to look after the PLS *(Arrow 6)* or by reduced effectiveness due to worry, inability to concentrate, or, as one caregiver put it, *‘the burden on my heart’ (Arrow 7).* In addition, most families had suffered considerable financial losses due to the costs associated with getting treatment, the PLS′ and other family members' reduced capacity to do paid work, or the financial burden of having to care for a female PLS who had never married or returned home after divorce.

Finally, several caregivers spoke about how their status in the community had been affected simply by being associated with the PLS, or being *labelled* as a member of a ‘mad house’ (*Arrow 2)* (Additional quotes Online File D).

Despite the prevalence of negative reactions, many caregivers also reported *‘supportive’* social responses, e.g., *financial help, advice, reassurance* and *practical help with looking after the PLS.*

##### Key theme 3: Caregivers' emotional reactions and feelings about the self

With some exceptions, most caregivers reported a great emotional burden as the result of the PLS’ condition. Some expressed their distress in moving accounts and were upset during the interviews.

Caregivers' reported emotions were dominated by an emphasis on *‘worry and tension’*. For example, caregivers were concerned about the PLS’ health, wellbeing and future, particularly their marital and employment prospects, and the impact of the illness on the future of other family members. Related to this was the constant worry about *‘others finding out’*. A few admitted they were scared of the PLS’ behaviour, especially physical violence.

Furthermore, caregivers expressed feelings of *‘frustration and anger’* towards the PLS, often triggered by having to look after them with very little support. In addition, some revealed *‘feelings of shame’* associated with the PLS’ appearance of behaviour in public, or simply having a mentally ill family member. A few spoke about feeling bad about themselves or having ‘*lost self-esteem’*.

Several caregivers described feeling ‘*sad’* or *‘hopeless’*, sometimes using strong expressions to describe their despair. Five caregivers said they had *‘wanted to be dead’* or had thought about ending their lives.

As outlined above, many salient emotional reactions reported by caregivers were linked to caregivers' and PLS’ reduced ability to meet role expectations: the family's financial security, the PLS’ future and wellbeing or other family members' future prospects (*Arrow 7*). The PLS’ behaviour and appearance was also an important source of anger or shame reported by caregivers *(Arrow 8)*. In fact, most of these feelings were attributed to behaviours such as *talking inappropriately or too loudly in public*, *exhibiting disruptive and aggressive behaviours*, *overspending money* or *being withdrawn and distant*.

Finally, the negative reactions which PLS and caregivers experienced from other people were among the most salient sources of caregivers’ distress and negative feelings *(Arrow 10)*. Some felt the illness and the associated loss of respect had permanently marked their lives:

*My life is over. What is there in my life now? He is not going to become normal. (…) We are not going to be respected by others.*

Female caregiver, wife of male PLS, Tamil Nadu

##### Differences by gender

A majority of caregivers (two thirds) were female, in keeping with the commonly observed preponderance of women in caregiving roles in India (see e.g (Sharma et al., 2016).). To examine whether stigma affected male and female caregivers differently, we compared the accounts of caregivers by gender. Overall, female caregivers seemed to be more closely involved in the care of the PLS, and were sometimes the only ones left in the family who were still in contact with the PLS. Even so, they were criticised by others for both the causation and persistence of the PLS’ ‘problem’, e.g. for not taking care well enough, having ‘ *delivered a mad person’,* or bringing an ill daughter-in-law into the house. Several women reported feeling shunned and avoided by neighbours and relatives, and some were concerned about their own marital prospects as a consequence of their association with the PLS.

Male caregivers, on the other hand, appeared generally more distant to the PLS, and possibly less isolated through stigma. The societal reactions they faced had to do with losing respect due to being associated with the PLS against their will, distance from friends and relatives, or societal expectations such as arranging marriage as a “cure” for the PLS. One male caregiver openly spoke about his deep frustration and resorting to beating his ill wife in anger when she did not fulfil her duties.

##### The wider context: caregivers' understanding of the illness and society's perceptions

When asked what they saw as their ill family members' problem (or reason for getting medical help), most caregivers used *descriptions of the behaviour that they saw as abnormal*, such as *‘roaming around’ or ‘speaking loudly’* in order to define it, e.g., “*The person who behaves below the normal person in the society […]* (Female caregiver, mother of female PLS, Tamil Nadu.

The behaviours and symptoms that were seen to set the PLS apart from others were sometimes called *‘behaving like a mad person’*. Whilst some caregivers explained that their family member had an illness that needed treatment, several others expressed doubts, and some specifically stated they did not see the behaviours exhibited by PLS as due to an ‘ailment’.

Most caregivers either expressed *uncertainty about the nature and cause of the PLS’ condition* or *attributed it to multiple causes simultaneously*. The causes cited most frequently *were ‘tension’/stressful life events’* (such as a bereavement, exam failure, or trauma), *‘evil spiriting/black magic’* (usually seen as the purposeful action of a third person) and *‘the behaviour of the PLS’ family members’*. For example, several caregivers thought that the condition might have been brought on by arguments in the family, or family members *‘spoiling’* the PLS, shouting at him or not loving him enough. A few caregivers blamed themselves for the illness, e.g. by attributing it to an extra-marital affair. Several others located the cause of the condition in the *‘the behaviour and characteristics of the PLS’*, for example in sexually inappropriate or isolative behaviour or the PLS' *‘adamant nature’.* Only a few caregivers cited *‘heredity’* or a ‘*bio-medical reason’,* such as the ‘brain not working properly’.

It was striking that many of the attributions listed above appeared to imply that someone had done something wrong or not put in adequate effort, and how these attributions were often accompanied by blaming or critical comments. A notable exception to this was the category of *‘tension/stressful life event’*.

Some caregivers spoke regretfully about how little they had known about the illness prior to the treatment, and how this had delayed help-seeking or affected the way they treated the PLS. They particularly lamented not knowing how to deal with the PLS’ unusual behaviour, which was often assumed to be intentional, and not knowing whether this was something that could be treated and would get better in the future.

*At first I thought that she was doing this all without any reason. (…) So we used to scold her. (…) Nobody suggested that we go and consult a doctor. (…) If I had brought her earlier [for treatment], then something could have been [done to make it] better. (…) I used to feel that if she had another type of disease then it could have been cured by medicines. But what treatment should there be for this illness? I did not know about it.*

*Female caregiver, mother of female PLS, Satara*

There was a widely held view among caregivers that this was *‘an illness that no-one should have’*, that it was *‘bad’* or, at the very least, difficult to manage. Some caregivers stated that it was *‘incurable’* or that it brought *‘dishonour’* and *‘different treatment’* to those affected.

Whilst only a small number of caregivers used the terms ‘stigma’ or ‘discrimination’ in their narratives, several offered descriptions of how people with mental illness were excluded or treated with disrespect, or held views such as *‘society has a problem with this illness’*. At the same time, only few caregivers voiced anger at the reactions of other people. Rather, some appeared to see their negative reactions as the understandable or natural consequence of the ‘different behaviour’ of the PLS.

## 4 Discussion

This study provides a rare opportunity to explore how family caregivers of people with schizophrenia experience stigma whilst also taking into account relevant contextual factors, using a large dataset from India. Reflecting the caregiving realities in India described by other studies (Sharma et al., 2016)., there was a marked female preponderance in our study sample (with 67% of caregivers being female).

### 4.1 Integrated descriptive findings on caregivers' experiences of stigma

‘High caregiver stigma’ was reported by a significant minority of caregivers: 21% had experienced stigma on each of the 10 domains covered by the scale in the last 12 months. Many more had experienced stigma in at least one domain (83%). Even though most PLS had been ill for several years (the median duration of illness was 6.3 years), many caregivers continued to worry about stigma and tried to hide the condition.

Qualitative interviews illustrate that for those caregivers experiencing stigma, its impact on relationships and emotional wellbeing was often very high. Particularly salient were experiences of being blamed, and critical comments and avoidance by others, which were linked to emotional distress, hopelessness and social withdrawal. Also important, and connected to worry and intrafamilial conflict, were concerns about ‘others finding out’ and its impact on relationships and marital prospects of the PLS and other family members. Notably, worries about what *might happen* (anticipated stigma), or *might be happening* (e.g., others gossiping or looking down upon the family; perceived stigma) and attempts to prevent loss of status for the whole family featured more prominently than the actual experience of negative reactions – as has been found for PLS’ experiences (Koschorke et al., 2014). The prominence of the abovementioned concerns is also reflected in the ranking of the caregiver stigma item scores, where worries about marital prospects (44%) and anticipated negative treatment from neighbours (40%) were most commonly endorsed (Fig. 1).

Less commonly reported were facets of internalised stigma, such as shame (34%) and self-blame (21%), however, those caregivers who did experience these reported them to be impactful. It was striking that many caregivers appeared isolated and unsupported and would avoid social interactions for fear of stigma.

Whilst caregivers reported stigma with similar or higher frequency than in other studies which have used this scale (Shibre et al., 2001, Thara and Srinivasan, 2000) it is still worth noting that many caregivers had low levels of stigma or none at all, which is in keeping with the findings of other relevant studies of caregiver stigma (Girma et al., 2014, Phelan et al., 1998, Phillips et al., 2002, van der Sanden et al., 2013). For example, whilst 45% stated they were uncomfortable to disclose the illness, a similar number (48%) were comfortable to speak about it. It is possible that factors such as the degree to which disclosure had already taken place (in rural settings and after years of illness possibly very high) might have influenced these findings. In addition to examining ways of reducing the negative impact of stigma onto caregivers, future research should seek to explore which factors may be protective against experiencing high stigma, and derive lessons for family interventions (Evans-Lacko et al., 2014, Rusch et al., 2009a, Rusch et al., 2009b). .

### 4.2 Integrated findings on factors influencing caregiver's experiences of stigma and negative reactions

Caregivers' experiences of stigma were closely linked to those of their ill family member and influenced by the same three key factors identified for the negative reactions faced by PLS: Symptoms of schizophrenia, ‘others finding out’ and reduced ability to meet role expectations (Koschorke et al., 2014).

#### Symptoms of schizophrenia and caregiver stigma (pathway I; marked green in Fig. 2)

Both in quantitative and qualitative data, a link was evident between the PLS’ behaviour and symptoms/illness manifestations and caregivers' experiences of stigma. Multivariate regression models of caregiver stigma (see Table 2 Online File B) show that caregivers of PLS with higher levels of positive symptoms of schizophrenia experienced higher levels of caregiver stigma. This mirrors earlier findings from this study, that PLS’ own experiences of negative discrimination were predicted by higher levels of positive symptoms (p = 0.05) and lower levels of negative symptoms(Koschorke et al., 2014).

In qualitative data, ‘positive symptoms’, particularly aggressive or disinhibited behaviour in public, but also certain negative symptoms, such as poor self-care or ‘not speaking’, were linked to negative reactions towards caregivers and feelings of shame (Fig. 2, *Arrows 4 and 8*). The extreme example of a caregiver who was asked by villagers to kill her own daughter who had behaved aggressively to her in public illustrates the high social importance of adhering to behavioural codes of conduct. Concerns about *‘Behaviours and illness manifestations’* were also salient because they influenced *‘Other people finding out’* and’ ‘*Reduced ability to meet role expectations’* (*Arrows 1 and 6*.)

The importance of behavioural manifestations of schizophrenia in shaping caregiver stigma, particularly ‘positive symptoms’ such as aggressive, suspicious or sexually inappropriate behaviour, has also been documented in other studies from India (Raguram et al., 2004, Thara et al., 2003) and China (Phillips et al., 2002).

#### ‘Others finding out’ and caregiver stigma (pathway II; marked orange)

On quantitative measures, many caregivers (45%) indicated that they were uncomfortable to disclose the PLS’ illness, and ‘*others finding out’* emerged as a salient qualitative theme. Caregivers often felt responsible for trying to keep the illness a secret in the interest of the family, and the associated worry and social isolation adversely impacted their self-esteem and emotional wellbeing (*Arrow 9*). The key reason for disclosure being of such concern was that it was seen to influence both the PLS’ and other family members' marital prospects and work (*Arrow 4*), with negative repercussions for the family's social standing and negative social reactions (*Arrow 5*). A small number of caregivers reported negative reactions directly as a consequence of *‘Others finding out’* (*Arrow 2*), for example being labelled and avoided as a member of a ‘mad house’. This experience of being socially devalued simply by close association with an ill relative can also be explained using the concept of ‘contamination’ put forward by Goffman and taken up in later studies on family stigma (Corrigan et al., 2006, Goffmann, 1963, Larson and Corrigan, 2008). However, only relatively few caregivers reported stigmatising reactions directly as a result of being associated with a relative known as a “mad person” (illness label) (Pathway II), and much more salient were fears of the negative impact of ‘others knowing’ on the family's ability to meet role expectations in key areas of social salience, such as marriage and work (*Arrow 2;* Fig. 2), which in turn was seen to lead to negative social consequences (*Arrow 5;* Fig. 2). Our data therefore suggest that the labelling processes which lie at the core of stigma theory (Link et al., 1989) exerted their detrimental effects largely through their negative impact on meeting role expectations in key areas of social salience, such as marriage and work.

#### Reduced ability to meet role expectations and caregiver stigma (pathway III; marked red)

The qualitative study findings also highlight a link between PLS’ and caregivers' ‘*reduced ability to meet role expectations’* in terms of marriage, work or financial standing, and the negative reactions PLS and caregivers experienced (*Arrow 5*). This is mirrored in the quantitative finding that PLS’ disability levels were associated with caregiver stigma (Table 2 Online File B). The cultural importance of marriage as a focal point of stigma-related concerns is also evident in the finding that worries about marital prospects emerged as the most highly endorsed item on the caregiver stigma scale (44%), and is in line with the findings of earlier studies from India (Hopper et al., 2007, Raguram et al., 2004, Thara and Srinivasan, 2000, Weiss et al., 2001).

In our publication on PLS’ experiences of stigma (Koschorke et al., 2014), we examined a theoretical notion put forward by Yang et al. (2007). which posits that a crucial condition for understanding the experience of stigma in different cultural contexts is to understand ‘what matters most’ for social and moral standing in that context. Based on our findings, we postulated that “what matters most to the moral status of PLS in India is to be able to meet gender-specific role expectations with regards to marriage and work, and adhere to codes of conduct in terms of socially acceptable behaviour”(Koschorke et al., 2014) p. 157. The accounts and experiences presented in this paper suggest that the salience of marriage, work and socially acceptable behaviour also held true for family members. Caregiver accounts suggest that some caregivers may have felt the importance of achieving ‘what matters most’ even more acutely than PLS, as they were trying to negotiate between an ill loved one, the family reputation and the demands of society around them.

As discussed in (Koschorke et al., 2014), the religious imperative of doing one's duties in life in accordance with dharma (rooted in Hindu philosophy and influential across religious groups) and the importance of achieving work and marriage in the context of poverty and inadequate health and social care systems may have added to the salience of this domain ((Koschorke et al., 2014, Loganathan and Murthy, 2011)). The PLS’ and other family members' inability to meet role expectations was often a source of family tensions as well as low self-esteem, shame and hopelessness amongst caregivers (*Arrow 7*). Caregivers described negative social reactions directly as a consequence of the PLS not meeting social role expectations (they were often blamed and criticised for it) or as a result of the overall loss of the family's social standing *(Arrow 5).* Lack of achievement in social domains that ‘matter most’ (Yang et al., 2007) therefore added other powerful labels to the pathway of stigma and social exclusion, similar to what has been found in previous research from India (Thara et al., 2003).

### 4.3 The relationship between caregivers' knowledge and understanding of the PLS' illness and their experiences of stigma and negative reactions

#### Integrated findings on knowledge about schizophrenia and caregivers’ understanding of illness

Knowledge about Schizophrenia, as measured by the KASI, was relatively low in the sample of caregivers taking part in this study, when compared to studies carried out in HIC settings (Barrowclough et al., 1987), but similar to a study among family caregivers of PLS in China (Li and Arthur, 2005).

This was supported by qualitative interviews, in which many caregivers spoke with regret about not knowing enough about the condition. In particular, they wanted to know whether this was a condition that was treatable, and to what extent their family member could get better. Many caregivers expressed uncertainty as to what had caused the PLS’ condition, and often held many explanatory models simultaneously, as has been found in other Indian studies (Charles et al., 2007). Of note, many caregivers' did not see the PLS’ unusual behaviour as something that could be explained as an ‘illness’, and often assumed that the PLS were acting intentionally, an observation that has been made in other studies on stigma in LAMIC (Mathias et al., 2015, Thara et al., 2003).

#### The relationship between knowledge about schizophrenia and caregiver stigma

Contrary to our hypothesis that caregivers with higher levels of knowledge of schizophrenia would experience less caregiver stigma, this study found no quantitative association between caregivers' knowledge and their experience of stigma (Table 3 Online File C).

Qualitative findings, however, illustrate several ways in which caregivers' understanding of illness was linked to their experience of stigma:

One such connection relates to *beliefs about the illness*: Many caregivers reported that they had felt that the condition was *‘incurable’* or that it brought *‘dishonour’* and *‘different treatment’* to those affected, which added to their distress and hopelessness, and their decisions not to tell others or seek help. Caregivers' *perceptions of other people's views of the illness* added to this: several felt that other people looked down upon those with the illness and their families (*perceived stigma*), and these negative reactions were considered ‘natural’ or understandable by some caregivers. Some narratives reflect how these perceptions were linked to caregivers' sense of shame, low self-esteem, anticipated negative reactions and concerns about ‘others finding out. Finally, several of the *causal attributions* cited by caregivers (e.g., purposeful evocation of evil spirits, volitional ‘misbehaviour’ by the PLS, the PLS' personality or past sins, inadequate care by other family members, or the caregiver's own failings) implied that someone was *to blame* for the appearance or persistence of the problem. Similarly, *being blamed by others* formed an important aspect of caregivers' and PLS’ experience of stigma in this study (Koschorke et al., 2014)suggesting that causal explanations attributing blame were held more widely in participants' social networks. Caregivers often faced critical comments from neighbours and other family members as a result of the PLS’ behaviour or persistent illness that implied that these could have been prevented with the right attitude or effort. At the same time, some caregivers were themselves critical of their ill family member, and what they saw as intentional ‘misbehaviour’, ‘laziness’, or ‘not putting in the effort’ required to get better, as has been found in earlier studies from India (Mathias et al., 2015, Raguram et al., 2004, Thara et al., 2003). This was consistent with PLS’ own accounts of their experiences (Koschorke et al., 2014).

Could it be that blaming responses in our sample were linked to limited ‘knowledge about schizophrenia?’ Or, more generally, to the lack of a widely accepted illness concept that would explain unusual behaviours or reduced functioning without anyone being at fault?

There is some evidence to support this hypothesis: The described tendency to attribute blame for mental illness and its symptoms to the person affected or their family has been observed in many settings, including India (Mathias et al., 2015). Furthermore, research from China has suggested that caregivers’ critical responses towards PLS were linked to attributions that PLS played a causal role in their own behaviour (Yang et al., 2004), consistent with attributional theory (Weiner et al., 1988). Whilst, to our knowledge, this has not been examined quantitatively for the Indian context, the findings of this study support the notion that criticism and negative reactions from caregivers were more common amongst caregivers who believed that the PLS had a choice about their behaviour.

Sociological research has described simultaneous positive and negative stigma-related effects of introducing illness labels to explain manifestations of mental illness (Link and Phelan, 1999), coining the term ‘labelling paradox’ (Perry, 2011). In the context of this study, this paradox might have manifested in the following way: with limited caregiver knowledge, stigma appeared to be primarily directed towards socially unacceptable behaviours, rather than a (psychiatric) illness label, as evident in descriptions of abnormal behaviour being used to define the PLS’ ‘problem’. This would have reduced the likelihood of being stigmatised via an illness label (*Pathway II;* Fig. 2) and given those affected by stigma the chance to recuperate their social standing once the PLS’ abnormal behaviour was back in control (*Pathway I;* Fig. 2). On the other hand, not being able to draw upon an illness concept that would explain abnormal behaviours without anyone being at fault, might have had negative repercussions in the form of blaming and critical comments. Supporting this, there was evidence, both in caregivers' and PLS’ qualitative narratives, that not understanding the symptoms of schizophrenia led to blaming responses. Some caregivers specifically said that learning that the PLS’ behaviours were due to an illness and not intentional helped them respond better, which had in turn improved the relationships within the family (see interview quotation above). Others reported that learning about the availability of treatment had been a huge relief and made it easier to talk to others about the condition.

Conversely, quantitative caregiver stigma scores (a conglomerate measure of caregiver stigma rather than blaming per se) were not correlated with knowledge about schizophrenia as measured by the KASI. As the KASI measures knowledge about schizophrenia according to a Western bio-medical model of mental illness, this suggests that having a better understanding of this biomedical illness model did not reduce stigma, at least not the aspects of stigma measured quantitatively. One possible reason for this might be that caregivers usually held several explanatory models and illness beliefs simultaneously, as has been found in other Indian studies (Charles et al., 2007, Das et al., 2006). That is, even if caregivers had some ‘knowledge’ in the bio-medical sense and reported this on the KASI questionnaire, they may have simultaneously held other views which had more bearing on their experience of stigma. This hypothesis would be supported by the findings of an earlier trial of an educational intervention in India which demonstrated that family caregivers held multiple explanatory models simultaneously only some of which were changed by the intervention (Das et al., 2006). It is also interesting that regression analyses on the experiences of PLS unexpectedly found that PLS whose caregivers had higher levels of knowledge about schizophrenia, experienced *higher* levels of negative discrimination (Koschorke et al., 2014). That is, caregivers being more aware of the bio-medical illness model of schizophrenia did not protect them from experiencing stigma, and possibly had a negative effect on PLS’ experiences of discrimination (the nature and direction of the association remain unclear). These findings are supported by a growing body of research indicating that certain forms of knowledge, particularly information projecting a biomedical model of mental illness, may increase rather than decrease social distance from people with mental illness (Phelan et al., 2006, Schomerus et al., 2012, Yang et al., 2012). Overall, our findings suggest that ‘knowledge about schizophrenia’ interacts with the process of stigmatisation in complex ways. They add to the existing evidence that knowledge conveying a bio-medical model of illness may not be beneficial or even harmful when seeking to reduce stigma. On the other hand, some aspects of knowledge, such as information on the nature and management of symptoms, may be helpful in terms of reducing certain types of stigmatising responses, e.g. blaming. Educational interventions need to consider carefully what messages should be conveyed in the interest of reducing stigma, taking into account context-specific illness beliefs and attributions (Charles et al., 2007, Das et al., 2006).

### 4.4 Study limitations

Despite efforts to make the study sample representative, it is limited to caregivers of PLS in psychiatric care. We have further discussed that the KASI reflects knowledge of a biomedical model of schizophrenia, and it is possible that results would have been different if a measure of illness understanding more in line with local explanatory models had been used. Language and cultural barriers, and the fact that many study collaborators were psychiatrists, may also have played a role in data analysis and interpretation. There was oversampling of dyads with higher discrimination reported by PLS in the qualitative sample, which might have increased the likelihood of more severe stigma in families being reported in the qualitative data. However, the impact on caregiver stigma findings overall is considered limited, as confirmed by a comparison of caregiver stigma scores between the total sample and the qualitative sample which revealed no significant difference (p = 0.10). Finally, it is possible that social desirability, loyalty and the wish not to speak negatively about family members may have influenced the findings.

### 4.5 Implications

The findings of this study have implications for research, practice and stigma interventions. Our findings illustrate that the impact of stigma on the lives of some family members needs to be recognised in the planning and implementation of anti-stigma interventions and health care interventions to support PLS in India. This is important to achieve improvements for PLS, but also a relevant outcome in its own right, given the enormous economic, social and emotional impact faced by family caregivers.

Based on the findings of this study and existing research, we would recommend that such interventions adopt a systemic approach that recognises the close links between PLS’ and caregivers' experiences and caregivers double role as people experiencing, and sometimes, enacting stigma (John et al., 2015, Phillips et al., 2002, Singh et al., 2016, Trani et al., 2015). Health services for PLS need to create spaces where caregivers can speak openly about their own experience of stigma and other needs (possibly using a locally adapted caregivers’ needs assessment (Wancata et al., 2006)). Respite help, contact with peer support groups, and opportunities to access healthcare, emotional and social support in their own right should be facilitated for caregivers where feasible and appropriate.

The data obtained from caregivers in this study support our previous recommendations that care interventions should focus on ‘what matters most’ (Yang et al., 2007) to people's sense of worth and social acceptance in their local context, particularly recovery-oriented work to support PLS’ taking up social roles that fulfil them and earn them respect, e.g. in marriage and work. Treatments to help PLS manage the types of illness manifestations most clearly associated with negative reactions, e.g. positive symptoms, socially unacceptable behaviour and poor self-care, may help reduce stigma-related stress for both PLS and caregivers (Koschorke et al., 2014). Given many caregivers' concerns about ‘others finding out’, interventions may further offer support with disclosure decisions (Corrigan and Rao, 2012).

Further research is required to identify and test strategies to support family caregivers with their own experiences of stigma, and help them support the PLS with theirs. Future studies of stigma would further benefit from having measures available that have the power to differentiate between different stigma pathways, e.g. stigma of behaviours, stigma of illness labels and stigma of inability to meet role expectations (see also pathways I, II and II in Fig. 2).

In addition, research should ascertain which messages should be conveyed in educational interventions seeking to reduce the impact of stigma in a range of settings and target groups (Clement et al., 2010).

The findings of this study suggest, for the context of India, that educational interventions to reduce stigma should emphasise: i) that PLS can recover and lead meaningful lives, including in the life domains that ‘matter most’ in the local context, (Clement et al., 2010, Yang et al., 2007); ii) that disruptive behaviour and other illness manifestations can be helped by the right kind of treatment and support; and iii) that such treatment and support is available (a requisite, which will first need to be met, of course). The findings indicate caution, however, with regard to interventions that concentrate on promoting bio-medical illness notions or attributions, and indicate that it may be more helpful to adopt a pragmatic approach focused on recovery and treatment. At the same time, specific messages linked to causal attributions, particularly *‘no-one is to blame’* may still be effective and need to be tested in interventions research.

The complex interplay of positive and negative reactions found in most narratives suggests that research should focus not only on how to reduce stigma, but also on how to enhance social inclusion and positive reactions (Mathias et al., 2015). Given the notable proportion of caregivers in this study who did not report high stigma experience, research should also examine factors determining stigma resilience and derive lessons for interventions.

Finally, anti-stigma efforts need to reach beyond healthcare interventions to achieve lasting changes in public attitudes and behaviours towards people with mental illness and their families, for example through community interventions (Sarkar and Punnoose, 2016, Thornicroft et al., 2016). Qualitative findings further indicate an urgent need to reduce the emotional and financial burden of caregiving and negative impact of stigma through structural changes, for example social and financial supports such as family pensions and disability benefits, and importantly, adequate and accessible healthcare for PLS and family members.

## Figures and Tables

**Table 1:** Key sample characteristics.

Characteristics of Caregivers	n (%)	Characteristics of PLS	n (%)
**Caregiver gender**		**PLS gender**	
Male	93 (33.0)	Male	150 (53.2)
Female	189 (67.0)	Female	132 (46.8)
**Caregiver age (years)**		**PLS age (years)**	
16–34	54 (19.2)	16–24	36 (12.7)
35–44	45 (16.0)	25–34	97 (34.4)
45–54	76 (27.0)	35–44	90 (31.9)
55–64	61 (21.6)	45–54	38 (13.5)
65 or above	46 (13.3)	55 or above	21 (7.5)
**Caregiver marital status**		**Symptom Severity (PANSS Scores)**	***Mean (SD)***
Married	215 (76.2)	PANSS Total Symptom Score (Possible range: 30–210)	75.7 (19.9)
Single	26 (9.2)	PANSS Positive Symptom Score (Possible range: 7–49)	17.5 (6.7)
Separated/Divorced	1 (0.4)	PANSS Negative Symptom Score (Possible range: 7–49)	21.4 (7.5)
Widowed	40 (14.2)	PANSS General Symptom Score (Possible range: 16–112)	36.9 (10.1)
**Caregiver occupational status**		**Level of Disability (IDEAS Total Score)** (Possible range: 0–20)	9.6 (4.5)
Not income-generating	119 (42.2)		
Income-generating	142 (50.4)		
Any other	21 (7.5)		
**Caregiver education level**		Household/Family characteristics	n (%)
Up to 5th Standard	109 (38.7)	**Highest education level in the household**	32 (11.4)
6^th^ - 8th Standard	47 (16.67)	up to 8th Standard	114 (40.7)
9th – 12th Standard	79 (28.0)	9th – 12th Standard College or above	134 (47.9)
College or above	47 (16.7)		
**Type of relationship to PLS**		**Family financial status**	
Parent	145 (51.4)	Living comfortably/Doing alright	86 (30.5)
Spouse	70 (24.8)	Just about getting by	78 (27.7)
Sibling	36 (12.8)	Finding it (very) difficult to make ends meet	118 (41.8)
Other family member	31 (11.0)		

## Appendix A Supplementary data

Web appendix Web appendix
