# Supplementary material for: Experiences of stigma and discrimination faced by family caregivers of people with schizophrenia in India
Source: Soc Sci Med. 2017 Apr;178:66–77. doi: 10.1016/j.socscimed.2017.01.061 (PMC5360174; doi:10.1016/j.socscimed.2017.01.061)
Supplement: Web appendix [file mmc1.docx]

**Online file A**

Table 1 Full sample characteristics

| **Characteristic** | **n(%)/ Mean (SD)** |
| --- | --- |
| **Number (%) of PLS – caregiver dyads participating in study** | 282 (100) |
| ***PLS characteristics*** | ***n (%)*** |
| **PLS Gender**  Male  Female | 150 (53.2)  132 (46.8) |
| **PLS Age (years)**  16 – 24  25 – 34  35 – 44  45 – 54  55 or above | 36 (12.7)  97 (34.4)  90 (31.9)  38 (13.5)  21 (7.5) |
| **PLS Marital Status**  Never married  Married  Separated/Divorced  Widowed  *Missing* | 121 (43.4)  121 (43.4)  23 (8.2)  14 (5.0)  *3* |
| **PLS Occupational Status**  Not income-generating (Unemployed; Housewife)  Income-generating (Paid employment; Self-employment)  Any other (e.g. student; retired)  *Missing* | 204 (73.1)  64 (22.9)  11 (3.9)  *3* |
| **PLS Education Level**  Up to 5^th^ Standard  6^th^ - 8^th^ Standard  9th – 12^th^ Standard  College or above  *Missing* | 64 (22.9)  61 (21.9)  112 (40.1)  42 (15.1)  *3* |
| **PLS Religion**  Hindu  Muslim  Christian  Other  *Missing* | 222 (79.6)  8 (2.9)  47 (16.9)  2 (0.7)  *3* |
|  | ***Median (IQ range)*** |
| **Duration of Illness (years)** | 6.3 (6.0 – 11.0) |
| *Missing* | *19* |
|  |  |
| **Symptom Severity (PANSS Scores)(Kay et al., 1987)** | ***Mean (SD)*** |
| **PANSS Total Symptom Score**  (Possible range: 30 – 210) | 75.7 (19.9) |
| **PANSS Positive Symptom Score**  (Possible range: 7 – 49) | 17.5 (6.7) |
| **PANSS Negative Symptom Score**  (Possible range: 7 – 49) | 21.4 (7.5) |
| **PANSS General Symptom Score**  (Possible range: 16 – 112) | 36.9 (10.1) |
|  |  |
| **Level of Disability (IDEAS Scores)** | ***Mean (SD)*** |
| **IDEAS Total Score**  (Possible range: 0 – 20) | 9.6 (4.5) |
| ***Characteristics of Caregivers*** | ***n (%)*** |
| **Type of relationship to PLS**  Parent  Spouse  Sibling  Other family member | 145 (51.4)  70 (24.8)  36 (12.8)  31 (11.0) |
| **Caregiver gender**  Male  Female | 93 (33.0)  189 (67.0) |
| **Caregiver age (years)**  16- 34  35 - 44  45 - 54  55 - 64  65 or above | 54 (19.2)  45 (16.0)  76 (27.0)  61 (21.6)  46 (13.3) |
| **Caregiver marital status**  Married  Single  Separated/Divorced  Widowed | 215 (76.2)  26 (9.2)  1 (0.4)  40 (14.2) |
| **Caregiver occupational status**  Not income-generating  Income-generating  Any other | 119 (42.2)  142 (50.4)  21 (7.5) |
| **Caregiver education level**  Up to 5^th^ Standard  6^th^ - 8^th^ Standard  9th – 12^th^ Standard  College or above | 109 (38.7)  47 (16.67)  79 (28.0)  47 (16.7) |
| **Caregiver Religion**  Hindu  Muslim  Christian  Other | 225 (79.8)  8 (2.8)  47 (16.7)  0(0.7) |
|  | **Mean (SD)** |
| **Caregiver Burden (Burden Assessment Schedule; BAS(Sell, 1998))**  (Possible range: 40 – 120) | 66.9 (13.4) |
| **Knowledge about Schizophrenia (Barrowclough et al., 1987)**  (KASI Total Score)  (Possible Range: 6 - 24) | 13.4 (2.7) |
| **KASI sub-scores**  **(Possible Range: 1 – 4)**  Knowledge about diagnosis  Knowledge about symptomatology  Knowledge about aetiology  Knowledge about medication  Knowledge about course and prognosis  Knowledge about management | 2.1 (0.6)  2.3 (0.7)  1.9 (0.6)  2.3 (0.8)  2.0 (0.8)  2.8 (0.9) |
| ***Family/Household Characteristics*** |  |
| **Research site**  Tamil Nadu  Goa  Satara | 105 (37.2)  92 (32.6)  85 (30.1) |
| **Urbanicity**  Rural  Urban | 195 (69.2)  87 (30.9) |
| **Caste**  Scheduled caste  Scheduled tribe  Other backward classes  None of the above  Don’t know | 104 (36.9)  6 (2.1)  73 (25.9)  66 (23.4)  33 (11.7) |
| **Family type**  Joint Family  Nuclear Family | 120 (42.6)  162 (57.5) |
| **No. of persons living in the family**  Up to three  Four or five  More than five | 87 (30.9)  122 (43.3)  73 (25.9) |
| **No. of persons < 18 living in the family**  Up to two  Three or four  Five or more | 169 (59.9)  74 (26.2)  39 (13.8) |
| **Highest education level in the household**  up to 8^th^ Standard  9th – 12^th^ Standard  College or above | 32 (11.4)  114 (40.7)  134 (47.9) |
| **Financial status**  Living comfortably/ Doing alright  Just about getting by  Finding it (very) difficult to make ends meet | 86 (30.5)  78 (27.7)  118 (41.8) |
| **Type of house**  Owned  Not owned (rented; other) | 250 (88.7)  32 (11.4) |
| **Type of toilet facility**  Own toilet  Shared toilet  No toilet facility | 177 (62.8)  30 (10.6)  75 (26.6) |
| **Source of drinking water**  Tap water (in the house)  Other water source (public tap, river, etc) | 162 (57.5)  120 (42.6) |

BARROWCLOUGH, C., TARRIER, N., WATTS, S., VAUGHN, C., BAMRAH, J. S. & FREEMAN, H. L. 1987. Assessing the Functional Value of Relatives' Knowledge about Schizophrenia: A Preliminary Report. *British Journal of Psychiatry***,** 1-8.

KAY, S. R., FISZBEIN, A. & OPLER, L. A. 1987. The positive and negative syndrome scale (PANSS) for schizophrenia. *Schizophr Bull,* 13**,** 261-76.

SELL, H. 1998. *The burden assessment schedule,* New Delhi, World Health Organisation, Regional Office.

Online file B

Table 2 Key sample characteristics qualitative sample (n=36 PLS-caregiver dyads)

| ***Characteristics of Caregivers*** | ***n (%)*** | ***Characteristics of PLS*** | ***n (%)*** |
| --- | --- | --- | --- |
| **Caregiver gender**  Male  Female | 12 (33)  24 (67) | **PLS gender**  Male  Female | 18 (50%)  18 (50%) |
| **Caregiver age (years)**  16- 34  35 - 44  45 - 54  55 - 64  65 or above  **Caregiver marital status**  Married  Single  Separated/Divorced  Widowed  **Caregiver occupational status**  Not income-generating  Income-generating  Any other  **Caregiver education level**  Up to 5^th^ Standard  6^th^ - 8^th^ Standard  9th – 12^th^ Standard  College or above | 8 (22)  5 (14)  6 (17)  13 (36)  4 (11)  27 (75%)  4 (11%)  0  5 (14%)  13 (36%)  21 (58%)  2 (6%)  12 (33%)  6 (17%)  7 (19%)  11 (31%) | **PLS age (years)**  16 – 24  25 – 34  35 – 44  45 – 54  55 or above  **Symptom Severity (PANSS Scores)**  PANSS Total Symptom Score  (Possible range: 30 – 210)  PANSS Positive Symptom Score  (Possible range: 7 – 49)  PANSS Negative Symptom Score  (Possible range: 7 – 49)  PANSS General Symptom Score  (Possible range: 16 – 112)  **Level of Disability (IDEAS Total Score)**  (Possible range: 0 – 20) | 8 (22%)  14 (39%)  8 (22%)  4 (11%)  2 (6%)  ***Mean (SD)***  71.0 (17.5)  17.2 (6.4)  18.5 (6.4)    35.3 (10.4)  9.0 (4.1) |
|  |  | ***Household/Family characteristics*** | ***n (%)*** |
|  |  | **Highest education level in the household**  up to 8th Standard  9th – 12th Standard  College or above | 2 (6%)  19 (53%)  15 (42%) |
| **Type of relationship to PLS**  Parent  Spouse  Sibling  Other family member | 20 (56%)  9 (25%)  5 (14%)  2 (6%) | **Family financial status**  Living comfortably/ Doing alright  Just about getting by  Finding it (very) difficult to make ends meet | 14 (39%)  13 (36%)  9 (25%) |

**Online File C**

Table 3 Crude and adjusted regression coefficients for factors independently associated with caregivers’ stigma experience

| **Characteristic** | ***Crude Coefficients*** | ***95% CI*** | **p** | ***Adjusted^4^***  ***Coefficients*** | ***95% CI*** | **p** |
| --- | --- | --- | --- | --- | --- | --- |
|  |  |  |  |  |  |  |
| **Positive symptoms of schizophrenia**^1^ | 0.10 | 0.05 - 0.16 | <0.001 | **0.09** | 0.03 – 0.15 | 0.003 |
|  |  |  |  |  |  |  |
| **Levels of disability^2^** | 0.16 | 0.07 - 0.25 | <0.001 | **0.12** | 0.01 – 0.22 | 0.04 |
|  |  |  |  |  |  |  |
| **PLS Age^3^** | -0.08 | -0.14 -0.02 | 0.006 | **-0.09** | -0.14 - -0.29 | 0.003 |
|  |  |  |  |  |  |  |
| **Highest level of education in the household** |  |  | 0.005 |  |  | 0.03 |
| up to 8^th^ Standard | 0.00 |  |  | **0.00** |  |  |
| 9th – 12^th^ Standard | 0.22 | 0.00 - 0.43 |  | **0.23** | 0.03 – 0.44 |  |
| College or above | -0.00 | -0.21 - 0.21 |  | **0.08** | -0.13 – 0.28 |  |
|  |  |  |  |  |  |  |
| **Research site** |  |  | 0.007 |  |  | 0.03 |
| Tamil Nadu | 0.00 |  |  | **0.00** |  |  |
| Goa | -0.12 | -0.27 - 0.04 |  | **0.11** | -0.07 - 0.29 |  |
| Satara | -0.25 | -0.41 - -0.09 |  | **-0.11** | -0.29 - 0.08 |  |
|  |  |  |  |  |  |  |

^1^ PANSS positive symptom score (PLS) as categorical variable (quartiles), fitted as a linear trend (confirmed with Likelihood ratio test)

^2^PLS’ IDEAS total score as categorical variable (quartiles), fitted as a linear trend (confirmed with Likelihood ratio test)

^3^PLS age as categorical variable (groups: 16-24; 25-34; 35–44; 45–54; 55 or above), fitted as a linear trend (confirmed with Likelihood ratio test)

^4^ adjusted for all other variables listed in the first column of the table

**Online file D**

Table 4 Crude associations between caregiver stigma experience (CSMS) and knowledge about schizophrenia (KASI)

| **CSMS (continuous) and KASI (categorical) – crude associations** |  |  |  |
| --- | --- | --- | --- |
|  | ***Regression Coefficient*** | ***95% CI*** | ***p*** |
| **KASI Total Score Subgroups:** |  |  | **0.67** |
| Quartile 1 (Scores 6 – 11) | 0.00 | 0.00 |  |
| Quartile 2 (Scores 12 - 13) | -0.03 | -0.22 – 0.15 |  |
| Quartile 3 (Scores 14 - 15) | -0.01 | -0.19 – 0.17 |  |
| Quartile 4 (Scores 16 - 24) | 0.08 | -0.12 – 0.28 |  |
|  |  |  |  |
| **KASI Binary Sub-scores**  **(Relatively high (score 3 or 4) versus relatively low (score 1 or 2) knowledge)** |  |  |  |
|  |  |  |  |
| Relatively high knowledge about diagnosis | -0.37 | -0.21 – 0.14 | **0.67** |
| Relatively high knowledge about symptomatology | -0.28 | -0.16 – 0.10 | **0.68** |
| Relatively high knowledge about aetiology | 0.09 | -0.10 -0.29 | **0.34** |
| Relatively high knowledge about medication | 0.09 | -0.04 – 0.22 | **0.16** |
| Relatively high knowledge about course and prognosis | 0.04 | -0.11 – 0.17 | **0.63** |
| Relatively high knowledge about management | 0.05 | -0.08 – 0.19 | **0.42** |
|  |  |  |  |

**Online file E**

**Additional quotes**

- Example of caregivers reporting being *‘treated without respect’***:**

*My friends would look at me with disgrace. They would not respect me. Even my relatives would 'not respect me. I may not get respect in my old work place. (…) When there is a tribunal in our area, I could not go and talk there. I would feel very embarrassed and I would stay at home.*

Male caregiver, brother of male PLS, Tamil Nadu

- On the link between *‘Reduced ability to meet role expectations’* and *‘Negative reactions’*

*“I have a son who is 30 years old. I am yet to get him married. Since there is a girl with illness in the family, how can I get him married? I feel very bad for this situation. (…)I[My sisters] have got their sons and daughters married. Whenever I meet them, they curse me that I have not discharged my duty properly.”*

Female Caregiver, mother of female PLS, Tamil Nadu

- On being ‘labelled’ as member of a ‘mad house’

*Others look at him [the PLS] with shame. They call him “loose”. When anyone asks for our address, people refer to our house as ‘mad house’. (…) They would identify me as the brother of a mad person.*

Male caregiver, brother of male PLS, Tamil Nadu

BARROWCLOUGH, C., TARRIER, N., WATTS, S., VAUGHN, C., BAMRAH, J. S. & FREEMAN, H. L. 1987. Assessing the Functional Value of Relatives' Knowledge about Schizophrenia: A Preliminary Report. *British Journal of Psychiatry***,** 1-8.

KAY, S. R., FISZBEIN, A. & OPLER, L. A. 1987. The positive and negative syndrome scale (PANSS) for schizophrenia. *Schizophr Bull,* 13**,** 261-76.

SELL, H. 1998. *The burden assessment schedule,* New Delhi, World Health Organisation, Regional Office.
